# Risk Factors for Love Addiction in a Sample of Young Adult Students: A Multiple Mediation Model Exploring the Role of Adult Attachment, Separation Anxiety, and Defense Mechanisms

**DOI:** 10.3390/bs14121222

**Published:** 2024-12-18

**Authors:** Eleonora Topino, Marco Cacioppo, Shady Dell’Amico, Alessio Gori

**Affiliations:** 1 Department of Human Sciences, Libera Università Maria Santissima Assunta (LUMSA), Via della Traspontina 21, 00193 Rome, Italy; eleonora.topino@gmail.com (E.T.); m.cacioppo@lumsa.it (M.C.); 2 Department of Health Sciences, University of Florence, Via di San Salvi 12, Pad. 26, 50135 Florence, Italy; shadiron@gmail.com; 3 Integrated Psychodynamic Psychotherapy Institute (IPPI), Via Ricasoli 32, 50122 Florence, Italy

**Keywords:** adult attachment, separation anxiety, defense mechanisms, love addiction

## Abstract

In certain situations, romantic engagement with a partner can have detrimental effects on an individual’s well-being and overall health, exhibiting features attributable to addictive behaviors. Considering the clinical significance of this phenomenon and its prevalence among adolescents and young adults, the objective of this study was to investigate the potential associations between some risk factors for love addiction in a sample of university students, with a specific focus on adult attachment, separation anxiety, and defense mechanisms. A total of 332 participants (M_age_ = 23 years; SD = 2.462) completed a survey consisting of the Love Addiction Inventory—Short Form, Relationship Questionnaire, Seven Domains Addiction Scale (Separation Anxiety domain), and Forty Item Defense Style Questionnaire. The data were analyzed using Pearson’s correlation, and a multiple mediation model was also implemented. Results showed that fearful attachment was significantly and positively associated with love addiction. Furthermore, this relationship was mediated by separation anxiety and neurotic/immature defense mechanisms too. These findings contribute to the existing literature on love addiction and provide valuable insights for future research and clinical practice.

## 1. Introduction

### 1.1. Background and General Aim

The term “love addiction” refers to a dysfunctional pattern of romantic relationships characterized by obsessive seeking of emotional connection, leading to significant distress and impairment in daily functioning [1,2]. Unlike healthy relationships, which foster mutual support, love addiction involves excessive, uncontrollable cravings for validation and emotional attachment. This often results in compulsive behaviors, such as constantly pursuing new partners or idealizing harmful relationships, accompanied by cycles of euphoria and emotional distress similar to substance addiction [1,3]. The negative consequences include emotional instability, anxiety, depression, and impaired decision-making, as individuals prioritize romantic obsessions over essential responsibilities [2,3]. Within the literature, various explanatory models and definitions of love addiction have been proposed [4,5,6]. In this regard, the application of Griffiths’ components model of addiction [7] to this phenomenon holds significant scientific relevance. According to this perspective, salience, tolerance, mood modification, relapse, withdrawal, and conflict could be conceptualized as descriptive dimensions of love addiction [8], and this is in line with previous evidence showing that love addiction shares many features of other behavioral or substance addictions (see Sussman [3] and Earp and colleagues [9] for reviews).

The prevalence of this phenomenon has sparked increasing interest in the scientific literature [10], and specifically, love addiction appears to be notably common among adolescents and young adults from different nationalities. Griffin-Shelley has repeatedly highlighted the significance of this addiction within adolescent populations [11,12,13], and considerable attention has also been directed towards university students [14]. Several studies have reported significant prevalence rates of love addiction among students and young adults in various countries, including the USA, Canada, Iran, and Spain [15,16,17,18,19]. These findings suggest that love addiction is a widespread phenomenon affecting a substantial proportion of young people globally, emphasizing the need for further research and intervention. Given the prevalence of the phenomenon, as well as its clinical relevance [10], the exploration of elements that may contribute to greater vulnerability appears of great relevance. In this regard, several risk factors related to the social (e.g., social support, isolation [20,21]) or familiar context (e.g., childhood trauma, family functioning [22]), as well as biological (e.g., brain’s reward system, neurocircuitry, biochemistry, and genetics [3]) or psychological (e.g., low self-esteem, low resilience [23,24]) have been investigated. The present study aimed to enhance this empirical framework to favor the understanding of love addiction and its related factors in young adults, by specifically exploring the role of adult attachment, separation anxiety, and defense mechanisms. Indeed, while the existing scientific literature has provided evidence supporting the individual associations between such variables and their relevance to addictions [10,25,26,27,28], to the best of the authors’ knowledge, no previous research has simultaneously examined the interplay of these variables in relation to love addiction among young adults.

### 1.2. Literature Review and Hypotheses

The present study investigates the relationships between fearful attachment, separation anxiety, defense mechanisms, and love addiction, grounded in the theoretical framework of attachment theory [29,30]. This perspective highlights the pivotal role attachment experiences in shaping relational behaviors and emotional regulation strategies throughout the lifespan. Building on this foundation, the study proposes a model in which separation anxiety and defense mechanisms mediate the relationship between attachment and love addiction. An integrated review of the literature and the theoretical rationale for this hypothesized model is provided below.

#### 1.2.1. Attachment and Love Addiction

John Bowlby [29,30] defined attachment as the innate human inclination to seek closeness and emotional connections with significant others. Individuals, both in childhood and adulthood, exhibit relational behaviors guided by Internal Working Models (IWMs) of the self and others. These models are largely shaped by early experiences with caregivers and are subsequently reflected in adult interactions (see Fraley [31] for a review). Indeed, Internal Working Models (IWMs) encode beliefs about the self in terms of perceived worthiness of love and care, as well as beliefs about others in terms of trustworthiness in providing care or posing threats [32,33]. In line with this conceptualization, Bartholomew and Horowitz [32] operationalized the construct by considering these two dimensions (i.e., the “Model of self” and “Model of other”), thus distinguishing four different attachment styles, one of which is secure (characterized by a good consideration of both the self and the other), and three insecure: (1) preoccupied, i.e., poor conception of the self and a good consideration of the other; (2) fearful, i.e., poor conception of both the self and the other; (3) dismissing, i.e., good conception of the self and poor consideration of the other. Previous evidence has emphasized the role of insecure attachment in contributing to psychopathology (see Mikulincer and Shaver [34] for a review), highlighting associations with mental health issues such as depressive symptoms [35], anxiety [36], and eating disorders [37], to name a few. Furthermore, the scientific literature has consistently demonstrated the influence of insecure attachment as a risk factor for the development of addiction [25,38], as well as its severity [39], and this evidence also extends to love addiction [9,10]. For example, Sussman [3] highlighted that individuals with insecure attachment may seek excessive closeness or become overly dependent on their partners—behaviors characteristic of love addiction. Additionally, Fisher et al. [40] examined the neurobiological underpinnings of romantic love and suggested that attachment processes play a significant role in the development of love addiction. Finally, Dineen and Dinc [41] recently confirmed the predictive value of insecure attachment for love addiction, further strengthening the evidence for this association. Among the different subtypes of insecure attachment, those characterized by high levels of anxiety (i.e., fearful and preoccupied) have attracted greater attention for their associations with love addiction [23,41]. However, research in this area remains limited and largely in its preliminary stages. Indeed, while findings on fearful attachment have been relatively consistent [42,43], more contrasting results emerge for the preoccupied pattern, which has sometimes been found to be uncorrelated with emotional dependence on a partner [44].

#### 1.2.2. The Potential Mediating Role of Separation Anxiety

Insecure attachment, particularly in its anxious forms, has been found to be associated with symptoms of separation anxiety [45,46]. Separation anxiety refers to the distress experienced in response to actual or anticipated separation from an attachment figure, serving as a natural mechanism that promotes closeness and security in early development [33]. However, when this anxiety persists and intensifies beyond early childhood, it may reflect challenges within the parent–child relationship, such as insecure attachment, potentially leading to broader adjustment difficulties in adult life [47,48]. Indeed, although separation anxiety was initially studied in childhood in the context of separation from the primary caregiver [29], there is an increasing body of scientific literature that recognizes the presence of separation anxiety in adulthood as well (see Bögels and colleagues [49] for a review), also including situations of separation from the partner [26].

Separation anxiety has been identified as a potential risk factor for mental illness [50] and may be a mediator in the relationship between attachment insecurity and maladaptive outcomes, including addictive behaviors. This condition has been associated with increased internalizing behaviors, poorer academic achievement, and compromised physical health [51]. Additionally, research highlights its role in fostering obsessive and compulsive relational behaviors, which contribute to dysfunctional patterns in interpersonal relationships [52]. Separation anxiety is characterized by persistent concerns about detachment from attachment figures or fear of being apart from them [53]. For individuals with anxious attachment, this heightened anxiety often manifests as an intense fear of abandonment, driving them to engage in clinging or excessively dependent behaviors, which are hallmarks of love addiction [54]. To mitigate these fears, such individuals may develop obsessive and compulsive relational behaviors, seeking constant reassurance and closeness. These behaviors, while attempting to address underlying fears of separation, frequently result in emotionally charged and dependent relational patterns that generate instability and distress for both partners. Consistent with this, evidence has shown that separation anxiety is negatively associated with both relational/marital satisfaction and mental health outcomes [55,56].

#### 1.2.3. The Potential Mediating Role of Defense Mechanisms

Attachment styles are associated with the use of defense mechanisms, which play a crucial role in regulating individuals’ responses to internal conflicts and emotional distress [57]. More specifically, defense mechanisms often emerge as responses to unresolved separation anxiety originating in early attachment experiences, where expectations of others as unreliable or rejecting are internalized [33]. These mechanisms operate unconsciously to mitigate anxiety and protect self-esteem, particularly when faced with threats to one’s self-concept or attachment bonds [57]. In general, defense mechanisms may help individuals to manage inner impulses and cope with external situations that are at odds with personal needs or desires [58,59,60,61], and may be distinguished into mature, neurotic, and immature mechanisms, based on the level of reality distortion involved in their use [62,63]. Consistently, research shows that insecurely attached individuals, particularly those with anxious patterns, are more likely to rely on immature or neurotic defenses [64,65]. Moreover, immature defense mechanisms may be used to cope with separation anxiety, and may exacerbate relational challenges by distorting perceptions of threats within relationships, thereby amplifying fears of separation and increasing relational difficulties [66]. Empirical evidence indicates that maladaptive defenses not only contribute to various psychopathological symptoms but may also serve as a pathway linking insecure attachment to relational maladjustment [67,68]. Consequently, insecure attachment can lead to defense styles that hinder emotional regulation, often manifesting in anxiety or interpersonal difficulties. While individuals with a mature defense style tend to report greater life satisfaction and overall well-being [69,70], reliance on neurotic and immature defenses has been linked to poorer mental health outcomes [57,71,72,73] and an increased vulnerability to both substance-related and behavioral addictions [27,74,75,76,77]. Addiction, including love addiction, has been conceptualized as an outcome of dysfunctional defensive strategies aimed at managing internal emotional distress through external means [78]. In this context, obsessive closeness to a partner may reflect an attempt to alleviate inner distress, often leading to relational instability. Consistently, interpersonal difficulties are frequently associated with the use of dysfunctional defense mechanisms [28].

### 1.3. The Present Research

Building on the above evidence, this study posits that separation anxiety and maladaptive defense mechanisms jointly mediate the association between fearful attachment and love addiction. Fearful attachment may foster separation anxiety and reliance on immature defenses, which in turn amplify dependency-driven relational patterns, culminating in love addiction. This integrated framework is consistent with theoretical and empirical models highlighting the interplay between attachment, emotion regulation, and addiction [3,10,41,53].

Based on the reviewed literature, the study hypothesizes the following:

**Hypothesis** **1:** *Fearful attachment* will be significantly and positively associated with *love addiction* (**H1**).

**Hypothesis** **2a:** *Fearful attachment* will be significantly and positively associated with *separation anxiety* (**H2a**).

**Hypothesis** **2b:** *Fearful attachment* will be significantly and positively associated with *neurotic* and *immature defense mechanisms* (**H2b**).

**Hypothesis** **3:** *Separation anxiety* will be significantly and positively associated with *neurotic* and *immature defense mechanisms* (**H3**).

**Hypothesis** **4a:** *Separation anxiety* will be significantly and positively associated with *love addiction* (**H4a**).

**Hypothesis** **4b:** *Neurotic* and *immature defense mechanisms* will be significantly and positively associated with *love addiction* (**H4b**).

**Hypothesis** **5:** *Separation anxiety* and *defense mechanisms* will significantly mediate the association between *fearful attachment* and *love addiction* (**H3**).

Finally, to account for the potential influence of gender and age on the variables of interest, previously identified in other studies [22,79], these factors were controlled as confounders. This step aimed to strengthen the validity of the hypothesized interactions within the model.

## 2. Materials and Methods

### 2.1. Participants and Procedure

A sample of 332 Italian young adults (80% Female, 20% Male), who declared themselves to have a romantic relationship, have been involved in this research. Their mean age was 23 years (*SD* = 2.462; range = 18–33 years). All of them were students who had been randomly invited to participate in this research voluntarily, through a snowball sampling method. The inclusion criteria were (1) being involved in a romantic relationship and (2) being at least 18 years old. The survey was administered online and was hosted on the Google Forms platform. Before starting, each participant was informed about the general aim of the study and provided informed consent electronically. All the procedures of this research have been approved by the first author’s institutional Ethical Committee.

### 2.2. Measures

#### 2.2.1. Love Addiction

The *Love Addiction Inventory—Short Form* (LAI—SF) [7] is a self-report measure used for evaluating the levels of love addiction. It consists of 6 items (e.g., *“Stay with your partner to relieve stress”*) elaborated following the components model of behavioral addiction [6], which are rated on a 5-point Likert scale from 1 (*Never*) to 5 (*Very often*). In this research, the total score of the short-form original (Italian) version [7] was used and showed acceptable internal consistency (*α* = 0.66).

#### 2.2.2. Attachment

The *Relationship Questionnaire* (RQ) [32,80] is a self-report measure used for evaluating attachment style, focusing on secure, fearful, preoccupied, and dismissing patterns. It consists of 4 items (e.g., *“It is easy for me to become emotionally close to others. I am comfortable depending on them and having them depend on me. I don’t worry about being alone or having others not accept me”*), which are rated on a 7-point Likert scale from 1 (*It does not describe me at all*) to 7 (*It very much describes me*). In this research, the Italian version [80] was used. Each attachment pattern was assessed with a single item, therefore the alpha coefficient of the scales cannot be calculated. Nevertheless, previous research showed that RQ presents good test–retest reliability [81] and good psychometric properties in different cultures [82].

#### 2.2.3. Separation Anxiety

The *Seven Domains Addiction Scale* (7DAS) is a separate self-report section of the Addictive Behavior Questionnaire (ABQ) [78] used for evaluating seven core domains associated with addiction disorders. Each domain consists of 7 items (e.g., “*Does it happen to you, when you are in love, that you are always afraid of being abandoned?*”) which are rated on a 5-point Likert Scale, from 1 (*Never*) to 7 (*Always*). In this research, the original (Italian) score of the Separation Anxiety domain [78] was used and showed good internal consistency (*α* = 0.85).

#### 2.2.4. Defense Mechanisms

The *Forty Item Defense Style Questionnaire* (DSQ-40) [63,83] is a self-report measure used for evaluating defense mechanisms, focusing on mature, neurotic, and immature patterns. It consists of 40 items (e.g., “*I get openly aggressive when I feel hurt*”), which are rated on a 9-point Likert scale from 1 (*Strongly disagree*) to 9 (*Strongly agree*). In this research, the Italian version [83] was used and showed acceptable internal consistency (Mature defenses: *α* = 0.62; Neurotic defenses: *α* = 0.67; Immature defenses: *α* = 0.77).

### 2.3. Data Analysis

The collected data have been analyzed by using the SPSS statistical software (v. 21.0, IBM, Armonk, NY, USA). As a preliminary analysis, the association between the variables was explored by performing a Pearson correlation. Then, a multiple mediation model was tested by including only the attachment pattern that showed a significant relationship with love addiction and by also testing the mediation of separation anxiety and defense mechanisms (mature, neurotic, and immature). The role of age and gender (coded with 1 = males, 2 = females) as confounders was controlled. Model 81 of the macro-program PROCESS 3.4 [84] was used to implement this analysis. To test the statistical stability of the models, the bootstrap technique (5000 bootstrapped samples with 95% CI, from boot Lower-Level Confidence Interval [Boot-LLCI] to boot Upper-Level Confidence Interval [Boot-ULCI]) [85] was used. Finally, post hoc power analyses were conducted using G*Power 3 software [86] to assess the achieved power for the mediation analyses given a sample size of 332 and an alpha of 0.05: a minimum power level of 0.80 is generally recommended in the social sciences [87,88].

## 3. Results

Pearson correlation (see Table 1) showed that love addiction was significantly and positively related to fearful attachment (*r* = 0.247, *p* < 0.01), separation anxiety (*r* = 0.312, *p* < 0.01), neurotic defenses (*r* = 0.254, *p* < 0.01), and immature defenses (*r* = 0.332, *p* < 0.01). The associations between love addiction and secure (*p* = 0.088), preoccupied (*p* = 0.061), and dismissing (*p* = 0.155) attachment patterns were non-significant.

**Figure 1:**
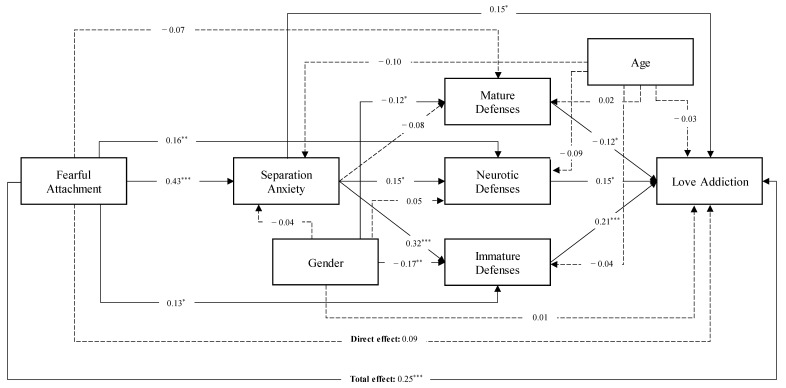
The Multiple Mediation Model. ***Note***: * < 0.05; ** < 0.01; *** < 0.001. Dashed lines represent non-significant associations; non-dashed lines represent significant associations.

On this base, a multiple mediation model was implemented, exploring the influence of separation anxiety and defense mechanisms (mature, neurotic, and immature) on the relationship between fearful attachment and love addiction (see Figure 1).

More specifically, a significant total effect was found in the relationship between fearful attachment and love addiction (*β* = 0.25, *p* < 0.001). The fearful attachment was also significantly and positively related to separation anxiety (*β* = 0.43, *p* < 0.001), which in turn was significantly and positively associated with neurotic (*β* = 0.15, *p* < 0.05) and immature defenses (*β* = 0.32, *p* < 0.001), but not with the mature ones (*β* = −0.08, *p* = 0.176). Furthermore, neurotic (*β* = 0.15, *p* < 0.05) and immature (*β* = 0.21, *p* < 0.001) defenses were significantly and positively related to love addiction. Concerning the covariates, being female was associated with both less mature (*β* = −0.11, *p* < 0.05) and immature (*β* = −0.16, *p* < 0.01) defenses. Finally, the direct effect in the association between fearful attachment and love addiction was non-significant, suggesting a total mediation (*β* = 0.07, *p* = 0.132): *R*^2^ *=* 0.182, *F*(7, 324) = 10.310, *p <* 0.001 (see Table 2).

Concluding, the bootstrap technique supported the statistical stability of the model, confirming the significance of both the total (Boot-LLCI = 0.240; Boot-ULCI = 0.642) and indirect (Boot-LLCI = 0.187; Boot-ULCI = 0.404) effects. The post hoc power analysis revealed excellent power for the Multiple Mediation analysis, with a value of 0.99.

## 4. Discussion

Love addiction represents a maladaptive and problematic phenomenon linked to the sentimental sphere, characterized by the continuation of the romantic relationship despite negative consequences [1]. Indeed, research in the scientific literature has revealed significant connections between love addiction and many negative outcomes, including depression [89], lower relationship satisfaction [90], and self-harm and suicidal ideation [91], among others. Considering the clinical significance of this phenomenon, investigating the risk factors for love addiction can provide valuable insights for targeted and effective interventions. Therefore, the present research aimed to explore the variables associated with love addiction in a sample of young adult students, with a specific focus on adult attachment, separation anxiety, and defense mechanisms.

### 4.1. The Association Between Attachment and Love Addiction

The relationship between adult attachment and love addiction was initially examined. The results highlight a single significant and positive association, i.e., the one with fearful attachment. The associations between love addiction and secure, preoccupied, and dismissing attachments were non-significant. Concerning the secure attachment pattern, although it is often recognized as an important protective factor for mental health (see Zhang et al. [92] for a review), it did not show significant relevance to love addiction in the present study, consistent with previous research [41]. This could suggest that attachment insecurity (more than security) could have a core role with respect to dependence in the emotional context. In this regard, aligning with previous studies [23], the dismissing attachment pattern was not significantly associated with love addiction. This may be attributed to the combination of low anxiety and high avoidance characteristics of this attachment style, which may lead individuals to avoid close relationships [32]. On the other hand, although both the preoccupied and fearful patterns present high levels of anxiety, only the latter showed a significant association with love addiction. Indeed, previous studies involving the general population have shown associations between high levels of attachment anxiety and love addiction [23]. However, other research has demonstrated that not only the anxious attachment dimensions of Need for Approval and Concern with Relationships are associated with love addiction, but also Discomfort with Closeness [93]. Consistently, fearful attachment is characterized by both high levels of anxiety and avoidance, as well as a negative view of oneself and others [32]. Such findings suggest that individuals with love addiction may not have an idealized view of their partners, but instead, they may overlook negative aspects of the relationship due to their fear of separation. These data have been further investigated through the implementation of the multiple mediation model, in which all the formulated hypotheses have been corroborated. Indeed, the total effect in the relationship between fearful attachment and love addiction was once again confirmed (**H1**), detailing and supporting the emphasis on the role of insecure attachment as a risk factor for both substance use disorders [94,95] and behavioral addiction [96,97,98].

### 4.2. The Associations Between Fearful Attachment, Separation Anxiety, Defense Mechanisms, and Love Addiction

Fearful attachment was also positively associated with separation anxiety (**H2a**), as well as with neurotic and immature defenses (**H2b**), while no significant relationship was found with the mature mechanisms. This is in line with previous evidence that suggests the potential distinction between attachment with high levels of anxiety and separation anxiety, although they are related to each other [99]. Indeed, attachment styles play a significant role in shaping social relationships and the expectations attached to them [93,100]. Adults with anxious patterns tend to experience hyperactivation of the attachment system, leading to heightened sensitivity to potential ruptures in their close interpersonal connections [34]. Furthermore, attachment is associated with the development and implementation of emotional management and regulation skills [30,78]. In this regard, defense mechanisms can be understood as mechanisms that modulate the attachment system to alleviate distressing emotions related to negative expectations [101,102]. Consistent with the results of this research, previous evidence showed that insecure attachment may hinder the implementation of mature defenses [101,103] and may be associated with higher use of neurotic and immature mechanisms [104].

Furthermore, separation anxiety was also significantly associated with defense mechanisms, specifically, with neurotic and immature defenses (**H3**). No significant relationships were shown with the mature ones. Indeed, defense mechanisms operate at an unconscious level to manage high levels of anxiety and distress arising from actual or perceived emotional threats, aiming to prevent them from entering conscious awareness [105]. However, in line with the findings of the present study, previous evidence highlighted that distressing interpersonal information may trigger the utilization of maladaptive defense mechanisms, particularly among individuals with insecure attachment styles [106].

Lastly, separation anxiety and defense mechanisms showed significant associations with love addiction (**H4a** and **H4b**). These findings align with existing research that has identified an excessive need for reassurance from the partner [107] and concerns about the relationship and its potential termination [108] among individuals who exhibit problematic patterns of romantic love, particularly those with high levels of obsessiveness (see Raffagnino and Puddu [109] for a review). Parallelly, the role of defense mechanisms in relation to mental health has been extensively shown in the scientific literature [110]. In this regard, previous studies highlighted the mediating role of dysfunctional defense mechanisms in the relationship between insecure attachment and psychological distress [64]. Furthermore, these data contribute to a growing body of research that supports the involvement of the defense mechanisms operating with higher levels of reality distortion or detachment from reality [58] in contributing to addictive behaviors [27,110].

### 4.3. The Mediation of Separation Anxiety and Defense Mechanisms

In conclusion, results showed that the relationship between fearful attachment and love addiction was totally mediated by the sequential effect of separation anxiety and neurotic/immature defense mechanisms (**H5**). These findings can be interpreted from the perspective of addiction as a strategy to externally regulate dysregulated internal emotional states [25,38,67]. Individuals with a fearful attachment style may internalize a self-view marked by feelings of unworthiness and a perception of others as unreliable or unavailable, often resulting in low self-confidence and fear of abandonment [93,100]. Such individuals frequently experience heightened separation anxiety and fearing rejection, which reinforces a cycle of dependency on external sources of emotional regulation [95,111,112]. Then, in the absence of the development of effective regulatory skills within the attachment relationship, individuals may resort to dysfunctional mechanisms for managing these negative emotional states, which may lead to an obsessive immersion in a romantic relationship with the partner [83,101,109]. In other words, they may turn to neurotic and immature defense mechanisms as coping strategies to suppress or avoid these painful feelings [57]. However, these maladaptive strategies rarely provide lasting relief, leading individuals to seek comfort and security in romantic relationships, where love addiction may manifest as an obsessive preoccupation with their partner [113,114].

### 4.4. The Confounding Effect of Age and Gender

The roles of age and gender were controlled as potential confounders in the models, consistent with previous research on behavioral addiction [115]. Neither variable was related to love addiction; however, gender was significantly associated with both mature and immature defenses. Specifically, females exhibited higher scores in both defense styles. This finding suggests that women may be more likely than men to use a broader range of defense strategies, encompassing both adaptive and maladaptive ones [116,117].

### 4.5. Limitations and Suggestions for Future Research

This research has several limitations that should be highlighted. First, given the incidence of love addiction in the adolescent and young adult population, this study was conducted involving university students, in line with previous research (see Sussman, Lisha, & Griffiths [14] for a review). Future research could benefit from including a more diverse sample, including individuals from clinical settings, to further explore and expand upon the results obtained in this investigation. Furthermore, the participants in this study were predominantly female (80%), which may restrict the generalizability of the results to males. The recruitment of more balanced samples in future research would help address this limitation and allow for a more comprehensive understanding of the phenomenon across genders. Moreover, the information collected about the participants’ family background is limited. Expanding data collection to include additional factors (e.g., whether participants come from single-parent families) could provide valuable insights and serve as a productive direction for future research. In addition, a cross-sectional design was adopted for this study. Therefore, although the direction of the associations between the variables studied is supported by the existing scientific literature, caution is needed in interpreting causal links. Future research should employ longitudinal approaches to retest the proposed model. Finally, only self-report measures were used in this study. While self-report measures have their advantages, such as providing insights into participants’ subjective experiences, they are also subject to potential biases and limitations, such as social desirability bias or memory recall biases. Therefore, future research could benefit from incorporating additional objective measures, such as behavioral observations or physiological assessments, to overcome this issue.

## 5. Conclusions

This study has shed light on the complex relationships between adult attachment, separation anxiety, defense mechanisms, and love addiction among young adults. By integrating these variables into a comprehensive model, the present findings provide a deeper understanding of the mechanisms underlying love addiction. The results highlight the role of insecure attachment, particularly the fearful pattern, as a significant risk factor for love addiction. The study also demonstrates the mediating role of separation anxiety and neurotic/immature defense mechanisms in this relationship. This suggests that individuals with a fearful attachment style may experience higher levels of separation anxiety, leading them to employ maladaptive defense mechanisms, which in turn contribute to the development or maintenance of love addiction. These findings are significant as they offer novel insights into the specific pathways through which attachment insecurity can lead to love addiction. By identifying the mediating effects of separation anxiety and defense mechanisms, the study advances the theoretical understanding and opens new avenues for research in this field. From a practical perspective, the significance of these findings lies in their potential to inform both future research and clinical interventions aimed at addressing this dysfunctional phenomenon among young adults. Indeed, the identification of these specific risk factors and mechanisms has important implications for therapeutic practice and intervention strategies. Targeted interventions that focus on addressing attachment-related issues, reducing separation anxiety, and restructuring maladaptive defense mechanisms may be effective in preventing and treating love addiction. Clinicians and therapists can utilize these insights to help individuals develop healthier coping strategies, enhance relationship satisfaction, and mitigate the negative consequences associated with love addiction.

## Figures and Tables

**Table 1:** Correlation Matrix.

	1	2	3	4	5	6	7	8	9
1. Love Addiction	1	−0.094	0.103	0.247 **	−0.078	0.312 **	−0.038	0.254 **	0.332 **
2. Secure Attachment		1	−0.359 **	−0.133 *	−0.165 **	−0.478 **	0.100	−0.066	−0.236 **
3. Preoccupied Attachment			1	0.185 **	0.156 **	0.518 **	−0.063	0.083	0.319 **
4. Fearful Attachment				1	−0.077	0.422 **	−0.091	0.207 **	0.275 **
5. Dismissing Attachment					1	0.103	0.109 *	−0.005	0.133 *
6. Separation Anxiety						1	−0.104	0.214 **	0.389 **
7. Mature Defenses							1	0.353 **	0.246 **
8. Neurotic Defenses								1	0.437 **
9. Immature Defenses									1
*M*	11.828	3.458	3.672	2.889	3.280	12.358	41.377	36.630	97.452
*SD*	3.154	1.896	1.913	1.785	1.716	6.219	8.211	8.372	21.744

***Note***: Bold values indicate significant *p* values. ** Correlation is significant at the 0.01 level (2-tailed). * Correlation is significant at the 0.05 level (2-tailed).

**Table 2:** Coefficients of the Multiple Mediation Model.

		M1				M2				M3				M4				Y		
		β	SE	*p*		β	SE	*p*		β	SE	*p*		β	SE	*p*		β	SE	*p*
X	*a*_1_	0.425	0.174	**<0.001**	*a*_4_	−0.068	0.278	0.265	*a*_8_	0.156	0.277	**0.008**	*a*_12_	0.128	0.671	**0.020**	*c*’	0.086	0.101	0.134
M1		-	-	-	*a*_5_	−0.082	0.080	0.176	*a*_9_	0.145	0.080	**0.014**	*a*_13_	0.322	0.192	**<0.001**	*b*_1_	0.146	0.030	**0.015**
M2		-	-	-		-	-	-		-	-	-		-	-	-	*b*_2_	−0.120	0.022	**0.036**
M3		-	-	-		-	-	-		-	-	-		-	-	-	*b*_3_	0.151	0.023	**0.013**
M4		-	-	-		-	-	-		-	-	-		-	-	-	*b*_4_	0.215	0.009	**<0.001**
C1	*a*_2_	−0.095	0.127	0.058	*a*_6_	0.0244	0.184	0.660	*a*_10_	- 0.090	0.184	0.694	*a*_14_	−0.037	0.445	0.468	*b*_5_	−0.029	0.066	0.579
C2	*a*_3_	−0.041	0.787	0.419	*a*_7_	−0.126	1.137	**0.022**	*a*_11_	0.047	1.134	0.381	*a*_15_	−0.165	2.749	**0.001**	*b*_6_	0.002	0.416	0.977
		*R*^2^* =* 0.188, *F*(3, 328) = 26.314, ***p <* 0.001**				*R*^2^* =* 0.031, *F*(4, 327) = 2.587, ***p <* 0.037**				*R*^2^* =* 0.074, *F*(4, 327) = 6.501, ***p <* 0.001**				*R*^2^* =* 0.193, *F*(4, 327) = 19.537, ***p <* 0.001**				*R*^2^* =* 0.182, *F*(7, 324) = 10.310, ***p <* 0.001**		

***Note***: Bold values indicate significant *p* values. Gender was coded as 1 = Male and 2 = Female; X = Fearful Attachment; M1 = Separation Anxiety; M2 = Mature Defenses; M3 = Neurotic Defenses; M4 = Immature Defenses; C1 = Age; C2 = Gender; Y = Love Addiction.

## Data Availability

The data presented in this study are available on request from the corresponding author. The data are not publicly available due to privacy reasons.
